# Polymer Kernels as Compact Carriers for Suspended Cardiomyocytes

**DOI:** 10.3390/mi14010051

**Published:** 2022-12-25

**Authors:** Mikhail Slotvitsky, Andrey Berezhnoy, Serafima Scherbina, Beatrisa Rimskaya, Valerya Tsvelaya, Victor Balashov, Anton E. Efimov, Igor Agapov, Konstantin Agladze

**Affiliations:** 1 Moscow Institute of Physics and Technology, Institutskiy Lane 9, 141700 Dolgoprudny, Russia; 2 M.F. Vladimirsky Moscow Regional Clinical Research Institute, Schepkina St. 61/2, 129110 Moscow, Russia; 3 Academician V.I. Shumakov National Medical Research Center of Transplantology and Artificial Organs, Ministry of Health of the Russian Federation, Schukinskaya St., 1, 123182 Moscow, Russia

**Keywords:** cell culturing, iPSC-CM, polymers, electrophysiological coupling, electrospinning

## Abstract

Induced pluripotent stem cells (iPSCs) constitute a potential source of patient-specific human cardiomyocytes for a cardiac cell replacement therapy via intramyocardial injections, providing a major benefit over other cell sources in terms of immune rejection. However, intramyocardial injection of the cardiomyocytes has substantial challenges related to cell survival and electrophysiological coupling with recipient tissue. Current methods of manipulating cell suspensions do not allow one to control the processes of adhesion of injected cells to the tissue and electrophysiological coupling with surrounding cells. In this article, we documented the possibility of influencing these processes using polymer kernels: biocompatible fiber fragments of subcellular size that can be adsorbed to a cell, thereby creating the minimum necessary adhesion foci to shape the cell and provide support for the organization of the cytoskeleton and the contractile apparatus prior to adhesion to the recipient tissue. Using optical excitation markers, the restoration of the excitability of cardiomyocytes in suspension upon adsorption of polymer kernels was shown. It increased the likelihood of the formation of a stable electrophysiological coupling in vitro. The obtained results may be considered as a proof of concept that the stochastic engraftment process of injected suspension cells can be controlled by smart biomaterials.

## 1. Introduction

Induced pluripotent stem cells (iPSCs) are now regarded as the most promising source of autologous cells for heart regeneration. Optimization of protocols for directed differentiation of the human iPSCs into ventricular-like cardiomyocytes (iPSC-CMs) made it possible to produce cells in sufficient quantities to develop methods of cell replacement therapy [1]. Intramyocardial injections of cells are of practical interest for targeted delivery of iPSC-CMs to damaged areas of the heart tissue to restore myocardial function. The feasibility of this approach has been shown in preclinical animal models. Intramyocardial injections of iPSC-CMs affect ejection fraction (PV-diagrams), cardiac wall thickness (histology), demonstrate epicardial-synchronous excitation (optical mapping) [2,3,4,5,6,7] However, electrocardiography (ECG) reveals regular engraftment arrhythmias (EAs), including transient ventricular tachyarrhythmias occurring 4–21 days after injection [3,8]. 

One of the features of iPSC-CM is the fetal-like phenotype, which causes cells to have no clear structure or shape due to the plasticity of the cytoskeleton [9,10]. Obtaining an iPSC-CM suspension from adherent cell monolayers alters the intracellular organization of its constituent cells: isolated cells are temporarily deprived of adhesion foci, contractile filaments order, and membrane excitability due to deprivation of contact with a solid substrate and neighboring cells. However, the plasticity of the cytoskeleton allows to restore the intracellular organization on a new substrate over time after intramyocardial injections, facilitated by the recipient’s myocardium [7,11].

Current methods do not allow one to control the processes of adhesion of injected cells to the tissue, the orientation of cells and the location of intercellular contacts in the new environment [12,13]. Therefore, important steps of iPSC-CMs engraftment into cardiac tissue, including restoration of intracellular organization and excitability, occur spontaneously, creating a risk of arrhythmias during early engraftment [8,14]. 

In this report, we isolated fetal-like iPSC-CMs and used polymer kernels to organize the contractile cytoskeleton and restore the excitability of the isolated cells at the time of injection. Polymer substrates of various architecture are widely used for tissue engineering for supporting cell conglomerates [15,16,17,18,19]. However, the common substrates with cultured cells forming patches of tissue are not easily suitable for low-invasive cell delivery, such as injection [20]. For this purpose, we used a minimized polymer substrate consisting of a single polymer fiber. We documented the possibility of the cell delivery process using fragmented polymer fibers (polymer kernels): biocompatible fiber fragments of subcellular size that can be adsorbed to a cell, thereby creating the minimum necessary adhesion foci to shape the cell and provide support for the organization of the cytoskeleton and the contractile apparatus prior to adhesion to the recipient tissue. The interaction of an isolated cell with a polymer fiber has previously been studied with confocal laser scanning microscopy (CLSM), scanning probe nanotomography and transmission electron microscopy [21]. Fetal-like CMs actively interact with substrate kernels, with creation of remarkable “sheath” spatial pattern enveloping the fiber. The fibers of our polymer kernels were pre-fragmented to subcellular sizes (precisely, subcellular diameter and linear length of the same order of magnitude as the length of a typical cardiomyocyte) and placed in a non-adhesive environment. Culturing isolated iPSC-CMs on polymer kernels restored intracellular order and excitability along with retention of mobility in suspension. 

To study the engraftment and electrical coupling of injected cardiomyocytes (with or without polymer kernels), a confluent monolayer of iPSC-CMs was used as a simplified model of cardiac tissue. Optical mapping with high spatial resolution was used to visualize the propagation of the excitation wave front, allowing to observe the correlation of excitations of the recipient monolayer and engrafted cells, in order to identify potentially arrhythmogenic cases. This work is aimed at studying the effect of pre-restored intracellular organization and cell excitability on the success of its electrophysiological coupling with the recipient tissue. Addressing this issue may be the key to a controlled process of single cell engraftment and a reduction in the risk of EA after intramyocardial injections.

## 2. Materials and Methods

### 2.1. The Processing of Silkworm Cocoons and Cell Cultivation on Microcarriers (Protocol 1)

The silk fibroin fibers were obtained from silkworm *Bombyx mori* silk cocoons, which were provided courtesy of Bogoslovsky V.V., the head of the State Scientific Institution of the Republican Scientific Research Station of Sericulture of the Russian Academy of Sciences (Zheleznovodsk, Stavropol region, Russia). At the first stage, cocoons were purified from sericin according to the following procedure. A 1 g by-weight portion of silk was boiled in 500 mL of double-distilled water containing 1260 mg of sodium bicarbonate in a water bath for 40 min. Then, the silk was washed with 3.6 L of distilled water. The silk was further boiled in 500 mL of double-distilled water in a water bath for 30 min and washed with 3.6 L of distilled water. The last procedure was repeated three times. The purified silk fibroin was dried at room temperature. Next, a portion of silk fibroin 80 mg by weight was incubated in a 3% aqueous solution of glycerol for 120 min. The silk fibroin was frozen in liquid nitrogen and ground for 5 min by mortar and pestle to an average fiber size of 200–1000 µm. The fibers were transferred into 5 mL of 70% ethanol aqueous solution and incubated for 30 min, and then the ethanol was changed. The obtained fibers were stored at 4 °C until use. Ultrastructure (SEM-image) of such a kernel is presented on Figure S1.

Next, the silk fibroin fibers were placed in a PBS buffered saline solution (pH = 7.4) containing 100 mg human fibronectin (HFN, Imtek). The process of HFN precipitation takes 24 h at physiological temperature. The seeding of dispersed iPSC-CMs on microcarriers was carried out as follows: fibroin kernels were kept in the HFN solution for 24 h, then subjected to centrifugation (200g, 5 min), the HFN solution was removed with a dispenser, the resulting sediment was about 10 thousand precipitated kernels. Next, 1 mL of cell suspension was added to the precipitated kernels (cell concentration is about 500 thousand per ml, RMPI1640 + B27 medium), the sediment was stirred in the suspension and completely transferred to a Petri dish (35 mm) with agarose coating of the bottom and walls (15 mg per 10 mL of 1X PBS, prepared and dried one day before adding the suspension) to avoid adhesion of cells to the side of plastic surface of the dish. After 24 h, attachment of cells to microcarriers was observed. 

### 2.2. Electrospinning of Aligned Polymer Fibers (Protocol 2)

The nano-scaffolds fabrication protocol was as follows. Cover slips with a diameter of 15 mm were covered with a 2% agarose solution in water. After solidification, the gel was dried at 70 °C to form a solid agarose film. Aligned electrospun nanofibers on parallel electrodes were located on the film surface. In the electrospinning process, a mixture of 2.5% poly-L-lactid acid (PLLA) and 10% collagen in a 10:1 ratio in hexafluoroisopropanol solvent was used. To prevent fiber detachment during the cell seeding process, they were coated with sucrose water solution (1g/1mL) by spin coating. Thereafter, the immobilized fibers on an agarose film were cut perpendicular to their main direction on a computer-controlled XY-table (Zeiss LSM 710). The glass with sucrose gel and fragmented fibers was then placed in a Petri dish (35 mm) with agarose coating of the bottom and walls (15 mg per 10 mL of 1X PBS, prepared and dried one day before adding the suspension). Then, 1 mL of cell suspension (concentration of 500 thousand cells per ml) was added to the dish, further cell sedimentation took 24 h. The approximate concentration of kernels per unit area was 500–600 microcarriers per mm^2^.

### 2.3. Cell Transfer Procedure

Cardiomyocytes were disaggregated using TrypLE Express (1X) (Sigma) to a unicellular state according to the following protocols [14]. The resulting cell suspension (1 million iPSC-CMs in 2 mL RRMI1640 + B27 medium) was stained with CellTracker Red, supplemented with 10 μM Y27632 (StemRD, USA) for survival experiments (Section 3.1) and optical mapping experiments (Section 3.3), suspension was divided into two equal groups. The first group was transferred (via injection with a 1 mL-dispenser) into a well with a formed iPSC-CMs monolayer (direct transplantation). The second group underwent the procedure of replanting (described above) with microcarriers and similarly transferred into the well with iPSC-CMs monolayer. We used the iPSC-CMs samples on the 60th day of differentiation as host monolayers.

### 2.4. Optical Mapping Protocol

According to the protocol described in [14], optical mapping with Fluo4 AM was performed in Tyrode’s salts solution (pH 7.25 to 7.4 units). The signal was recorded with a sampling frequency of 10 frames per second (Olympus DP72). The duration and amplitude of electrode stimulus were 20 ms and 6 V. The stimulation period was 1000 ms, unless otherwise noted (60 ppm). Data processing is described in Appendix A.

### 2.5. Immunofluorescent Staining

The protocol used for fixation and immunocytochemistry of the samples was made according to the recommendations from https://www.abcam.com/protocols/immunocytochemistry-immunofluorescence-protocol. Immunofluorescent staining was performed as previously described [14] on Zeiss LSM 710 confocal microscope with Zen black 3.0 software (Zeiss). 

Three-dimensional images were obtained as Z-stacks. Polar transformation of confocal images was performed with a Polar Transformer plugin in ImageJ (https://imagej.nih.gov/ij/plugins/polar-transformer.html). When performing the polar transformation, we used a polymer fiber as the main axis. The principle of transformation can be easily illustrated by analogy: transforming a globe around an axis of rotation will give an object on the surface of which there will be a flat map of the Earth. Calculation of volume (U) and cross-sectional area (A_xy_) was carried out in the ImageJ program: to calculate the U/A_xy_ ratios, we represented the surface of each cell/cluster as H (x, y)—a continuous set of points above the glass plane XY (Equations (1) and (2)).

(1) $$H\left(x,y\right):\;A_{xy}=A\left(H_{xy}\right)-cluster\;projection\;area\;on\;the\;XY\;plane$$

(2) $$U=\oint^{\;}H\left(x,y\right)dxdy\approx \;\sum^{\;}A*z^{\prime }\;\;sum\;of\;the\;areas\;\left(A\right)\;at\;each\;confocal\;slice\;\left(z^{\prime }\right)\;$$

### 2.6. Data Processing and Statistical Analysis

Statistical significance of differences between groups determined using a one-way ANOVA test followed by Fisher’s least significant difference test for group comparison; the differences were considered significant at *p* < 0.01 or statistically insignificant if *p* > 0.05.

To build a statistical model, a Gaussian curve approximation (to estimate μ and σ values) was used (Python 3, NumPy, sklearn and PyLab libraries). Assumptions underlying the statistical model can be founded in Appendix B. When using the statistical model, we considered the hypothesis to be statistically significant at *p* < 0.001, (Appendix B). Other calculations are given with an accuracy of one decimal place. A statistical model test was applied where it was impossible to apply one-way ANOVA test (please see Figures 5F and 6E caption).

### 2.7. Cell Culture and Ethical Approval

In this study, we used an m34Sk3-induced pluripotent stem cell line reprogrammed from a healthy (without hereditary cardiovascular diseases) donor [22]. The direct cardiac differentiation (ventricular-like iPSC-CMs) was performed in 24-well plates (covered with Matrigel) according to the modification of the Gi-Wi protocol [23,24]. Immunocytochemistry, optical mapping and flow cytometry have confirmed stable differentiation efficiency for the m34Sk3 cell line (50% of ventricular-like cardiac cells in population from 20 to 86th day of differentiation) [14,22,23,24]. The preparation of neonatal rat ventricular myocytes (NRVMs) samples was performed according to the Worthington protocol [19] (http://www.worthington-biochem.com/NCIS/default.html).

The iPSCs line m34Sk3 was created and provided by the E. Meshalkin Novosibirsk Scientific Research Institute of Circulation Pathology with informed consistent [22], handling was approved by the Institute of Circulation Pathology Ethics Committee (#27, 21 March 2013). All experiments and procedures were performed according to the principles for human experimentation as defined in the 1964 Declaration of Helsinki and its later amendments. All applicable international, national, and/or institutional guidelines for the care and use of cell lines were followed. Studies with NRVMs were conducted in accordance with the National Institutes of Health Guide for the Care and Use of Laboratory Animals (NIH publications No. 8023, revised 1978) and approved by the Moscow Institute of Physics and Technology Life Science Center Provisional Animal Care and Research Procedures Committee, Protocol #A2-2012-09-02.

## 3. Results

### 3.1. Polymer Kernels for Intracellular Organization Recovery

**Figure 1:**
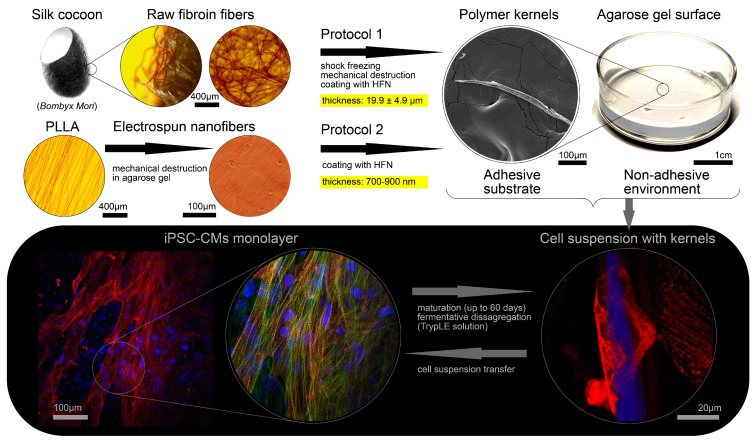
A schematic overview of the experiment design. The upper part represents protocols for obtaining polymer kernels and non-adhesive environment for their incubation with cell suspension. The lower part on a dark background shows the production of suspension iPSC-CMs with adsorbed polymer kernel and pre-restored cytoskeleton. Immunostainings are shown in pseudo colors: red for α-actinin, green for F-actin, blue for DAPI.

We used subcellular-sized polymer kernels to pre-restore the excitability and contractility of cardiomyocytes in suspension in order to influence the engraftment process into an excitable tissue (Figure 1). We have developed two methods of fiber fragmentation that allowed us to construct polymer kernels from both natural polymeric material (*Bombyx mori* silkworm cocoons, Protocol 1) and electrospun aligned nanofibers (Protocol 2). In Protocol 1 (Figure 1), purified silk fibroin was subjected to shock freezing and mechanical destruction to obtain a suspension of fibers with an average thickness of 19.9 ± 4.9 μm (average length of 452 ± 125 μm, n = 79). Next, the fibers were placed in PBS (pH = 7.4) solution containing human fibronectin (HFN); the difference in isoelectric points ensures the deposition of fibronectin on the surface of the fibers. Protocol 2 (Figure 1) consisted of sequential electrospinning of the poly L-lactic acid (PLLA) onto an agarose gel, cutting the gel using a computer-controlled XY-table (Zeiss LSM 710), and transferring the obtained polymer kernels into a HFN solution to create an adhesive surface (length 88.2 ± 6.2 μm, thickness < 900 nm, n = 50).

The ventricular-like iPSC-CMs were differentiated according to Gi-Wi protocol with subsequent 60-days maturation. Single cell suspensions were obtained from cell monolayers using TrypLE solution. The adhesion of single cardiomyocytes to the surface of polymer kernel was achieved by incubation in a non-adhesive environment during 24 h (Figure 1).

**Figure 2:**
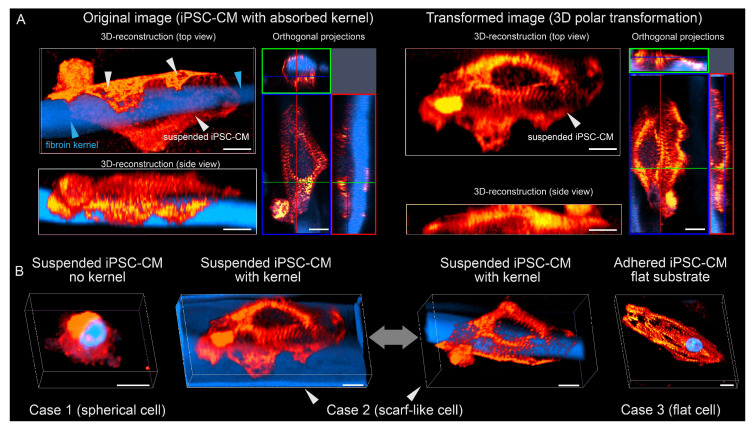
(**A**) Polar transformation of a 3D image of suspension iPSC-CM with adsorbed polymer kernel: original image is on the left; resultant image is on the right. Scalebar: 10 μm. (**B**) Three-dimensional image of iPSC-CM in suspension without a polymer kernel (right), an iPSC-CM in suspension with a polymer kernel (2 images in the middle), and iPSC-CM adhered to a flat substrate (left). Scalebar: 10 μm. Immunostainings are shown in pseudo colors: red for α-actinin, blue for DAPI.

To characterize the organization of the cytoskeleton of isolated ventricular-like iPSC-CM, we performed CLSM with α-actinin antibodies. Visualization of α-actinin shows the transverse banding required by the contractile cytoskeleton of myocytes. An example of transverse-banded cytoskeleton is visualized in Figure 2A based on a volumetric image of iPSC-CM on a fibroin microcarrier. We identified three possible options shown in Figure 2B: (1) an isolated suspension cell with a spherical shape without transverse banding (observed after incubation in non-adhesive environment in the absence of a polymer kernels); (2) an isolated cell with a scarf-like structure around polymer kernel; and (3) a flat cell on a full-fledged substrate with expressed transverse banding of α -actinin cytoskeleton. Figure 2A shows a polar transformation (centered on the polymer fiber axis) of a three-dimensional image of a cell with a polymer kernel. The resultant image shows the presence of pronounced adhesion foci on the surface of the polymer fiber and transverse banding of α-actinin cytoskeleton. Thus, cases 2 and 3 are equivalent to each other in terms of intracellular order and differ from case 1 by the presence of transverse banding and cell shape.

**Figure 3:**
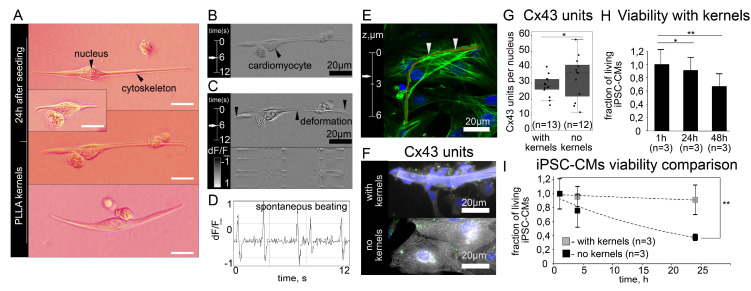
Suspended cardiomyocytes with adsorbed polymer fibers. (**A**) Seeding of suspended cardiomyocytes (NRVMs) onto PLLA fibers, collage, scalebar: 20 μm. (**B**) Isolated NRVM with adsorbed fiber. (**C**) phase map of spontaneous mechanical contractions, space-time plot and graph of optical density (**D**). (**E**) Confocal microscopy of a single NRVM with PLLA fiber after transfer to NRVM monolayer (F-actin shown in green, DAPI in blue, Rhodamine 6G in red). (**F**,**G**) Number of gap junction clusters identified by staining for connexin 43 in suspended iPSC-CMs with and without fibroin fibers (n = 3, * *p* > 0.05, One-way ANOVA test), α-actinin shown in grey, DAPI in blue, Cx43 in green. (**H**,**I**) Comparison of iPSC-CMs survival rate with and without fibroin fibers (n = 3, * *p* > 0.05, ** *p* < 0.01, one-way ANOVA test).

Since in our protocol (Section 2.1, Section 2.2 and Section 2.3) suspended cells and kernels could pin to each other, but not with immovable elements (walls or bottom of a Petri dish), they could be individually transferred to suspension without enzymatic disaggregation. An isolated single cardiomyocyte (NRVM) with adsorbed kernel is shown on Figure 3A,B. Spontaneous mechanical contractions were observed at 24 h after joint incubation (Figure 3C,D). Transfer of the cell with kernels to another dish was possible with a syringe or dispenser. Figure 3E shows a polymer kernel with adhered NRVM 48 h after transfer to the host NRVM monolayer, respectively (Section 2.2 and Section 2.3). Figure 3F,G show a comparison of the number of gap junction clusters identified by staining for connexin 43 in suspended iPSC-CMs with and without fibroin fibers (n = 3, * *p* >0.05, one-way ANOVA test, no statistically significant differences).

To test the long-term viability of suspended cardiomyocytes in the presence of polymer kernels and a possibility for secondary adhesion, we carried out the following experiment: at certain time points after isolation (from 0 to 48 h, Figure S2) we transferred dispersed cells with or without kernels onto HFN-coated glass with fresh culture medium. The next day, cells attached to the glass were observed and samples were fixed for immunocytochemical studies. Polymer kernels kept the population of viable adherent cells in suspension: the difference between the numbers of adherent cells was insignificant (n = 3, *p* > 0.05) within 24 h (Figure 3H). Figure 3I shows the difference in the number of α-actinin positive cells adhered to HFN-coated glass in studied groups. The suspension without polymer kernels was rapidly losing viable cells (Figure 3I). Finally, finding cardiomyocytes (iPSC-CM with polymer kernel) in an excitable state was possible for 96 h, which was verified using optical mapping (Figure S3). Thus, both NRVMs and iPSC-CMs restored excitability after adhesion to the polymer kernels and demonstrated that their phenotype was very similar to flattened cells on a flat substrate.

### 3.2. Co-Culturing of Excitable Cells on Polymer Kernels with a Monolayer of Cardiomyocytes

**Figure 4:**
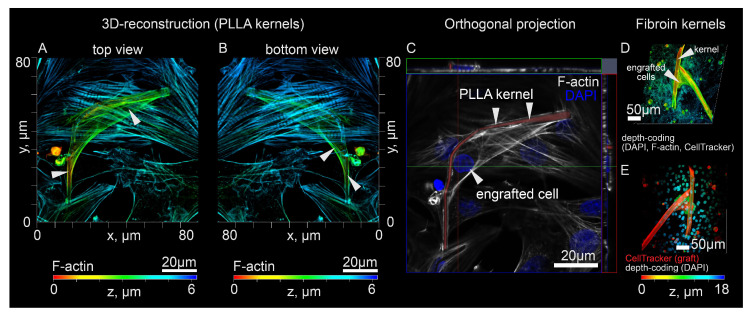
Three-dimensional reconstruction of cardiac monolayer after cell transfer with polymer kernels. (**A,B**) Depth-coding image (F-actin, DAPI) of NRVM monolayer with single NRVM graft (PLLA kernels). (**C**) Orthogonal projections, Rhodamine 6G shown in red, DAPI shown in blue, F-actin cytoskeleton shown in grey. (**D**) Depth-coding image (F-actin, DAPI, CellTracker Red) of NRVM monolayer with NRVM graft (fibroin kernels). (**E**) Depth-coding image (DAPI), CellTracker Red shown in red.

Since the developed cell culture approach could be applicable to cell transplantation via myocardial injection, we examined the final arrangement of the monolayer, polymer kernel and engrafted cells 48 h after contact. Upon contact with the host tissue, the scarf-like cell should form new adhesion foci during engraftment. Using CLSM, we found that in the graft, the polymer kernel was seen as an “internal” part, while the cell membrane was seen as an “external one”, with cells tending to wrap around the entire accessible surface of the fiber. Thus, engraftment did not lead to the destruction of the scarf-like structure, while the polymer kernel remained inside ("internal” part) the cell mass of the graft. Figure 4 demonstrates the reciprocal location of the engrafted cell, polymer kernel and the host tissue in greater detail. Therefore, the transferred cell’s membrane is brought into direct contact with the monolayer during engraftment without fiber interference (Figure 4A–E).

Supplementary Videos S1 and S2 demonstrate the mechanical connectivity of the resulting system where polymer fibers can be deformed both from the grafting cell’s forces and the monolayer contractions. However, a stable position of the graft was achieved with fibroin microframeworks; neither graft internal forces, nor host contractions were sufficient to deform and move engrafted cells (Video S3). Figure 4D,E demonstrate the engrafted NRMVs with fibroin kernels with the interaction between graft and host monolayer occurring directly. Altogether, initial mechanical stability and direct cell contact created the necessary conditions for the co-culturing of excitable cells (from monolayer) and excitable transferred cells on adsorbed polymer kernels. Based on this, we put forward a hypothesis that this approach to cell cultivation could influence the stochastic process of engraftment and the formation of intercellular contacts (for example, by the coculturing mechanism).

### 3.3. Evidence of Electrical Coupling between Isolated iPSC-CMs and Excitable Monolayer in Optical Mapping Experiments and Statistical Validation

Next, we experimentally tested the hypothesis that the presence of polymer kernels could influence the stochastic engraftment process and formation of intercellular contacts. Experimental verification consisted in a direct comparison of the total area of grafts with electrophysiological activity during implantation with a suspension and microcarriers (Section 2.1 and Section 2.3) and without them. Comparison was made in terms of the number and area of engrafted cells, measured by video recording of the Fluo-4 AM fluorescent dye, which changes intensity upon cell excitation (Ca^2+^ release into the cytoplasm). Below is a statistical model for the analysis of the experiment, followed by a detailed description of optical mapping and its analysis.

The structure of a mechanically stable graft and its location in the host tissue depend on the size of the cell, its structural and adhesive features, and also on the time elapsed after transplantation [25]. We assumed that the area of each graft obeys a normal distribution. Approximate values μ = 1721 (expected value of graft area, μm^2^) and σ = 711 (standard deviation, μm^2^) were estimated for confocal images of adhered iPSC-CM fixed at 48 h after transplantation. Data for the statistical model were obtained from four independent differentiations (m34Sk3, Gi-Wi protocol, n = 184 cells). The data on the area distribution formed the basis of a statistical model for comparison of the transfer efficiency by the total area of the detected grafts in accordance with its random and systematic differences (Equation (3)). In brief, m is the number of registered grafts (statistical sample size); μ, σ, n are the model parameters (see Appendix B).

(3) $$P_{\mu ,\sigma ,n}\left(m\right)={\left[\underset{a}{\max}\left(P\left(A|B\right)\right)\right]}^{m}\;\;\left(for\;\mu =1721,\;\sigma =711\right)$$

This statistical model takes into account both the number of excitable grafts m and the number of monolayers in each group (Appendix B). That allows us to compare area distributions of grafts visualized in optical mapping experiments.

**Figure 5:**
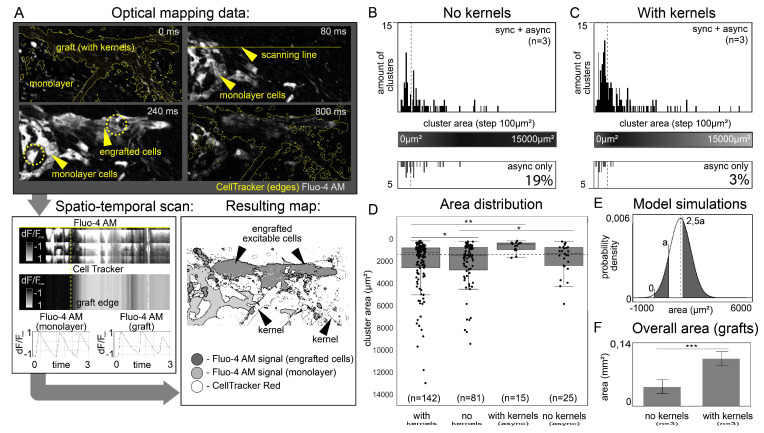
Distribution of excitable grafts by size (area). The dotted line shows the average area of a single isolated iPSC-CM after engraftment. (**A**) An algorithm for visualization and analysis of engrafted cells in the host tissue based on optical mapping. Black and white color shows the intensity of fluo-4 AM (spread of excitation through cells). Yellow line separates monolayer cells (initiator of excitation) from graft cells (receiver of excitation). The continuous conduction of excitation is illustrated by the spatio-temporal scan along the scanning line and Ca2+ traces (bottom left) plotted over the areas within the yellow dotted line. Resulting map shows the cell boundaries of the monolayer and graft, the area of the latter was used for panels B and C. (**B,C**) Diagrams showing the area distribution of engrafted cells. Grafts whose electrophysiological activity was asynchronized with the host are shown in the lower diagrams. (**D**) Area distribution of engrafted cells, * *p* > 0.05, ** *p* < 0.01. (**E**) Statistical model for comparing the overall areas of engrafted cells within groups, n = 2.5. The selected value of the parameter a maximizes P(A|B). (**F**) Diagram showing the difference in the overall areas of engrafted cells within groups (n = 3, *** *p* < 0.001 in statistical model).

In optical mapping experiments, two fluorescent labels were used, Fluo-4 AM calcium-sensitive dye to visualize excitation waves passing through the engrafted cells and the host monolayer, and CellTracker Red dye to distinguish and highlight boundaries between transferred cells and monolayer. Cumulative analysis made it possible to separate the boundaries and timing of wave propagation along the engrafted cells and the host monolayer (Figure 5A and Appendix A).

In each experiment, a freshly prepared iPSC-CMs suspension (marked with CellTracker Red dye) was divided into two equal parts for subsequent transfer to two host monolayers. The first half was transferred with the use of fibroin kernels (Protocol 1), and the second one was transferred without them. Optical mapping procedure was performed with periodic electrode stimulation of the host monolayer 48 h after cell transfer to register functional engrafted cells (Video S3). Figure 5B–D display the total number of registered grafts and their area distribution. Cases when electrophysiological activity of engrafted cells did not coincide with the period of monolayer stimulation are given separately (as asynchronized grafts) on the lower parts of panels 5B and 5C, and the right part of 5D. 

Size distribution (Figure 5D) revealed two important details: the overall distribution of graft’s area did not depend on the presence of fibroin kernel (*p* > 0.05), whereas the distribution of asynchronous grafts with polymer kernels was different (*p* < 0.01). In addition to increasing the total number of functional engrafted cells (Figure 5F), polymer kernels have reduced the proportion of asynchronized grafts (arrhythmogenic host-graft coupling) according to Figure 5B,C (3% for cells w/ kernels versus 19% for cells w/o kernels, n = 3).

Our null statistical hypothesis here is that the number of engrafted cells is the same in both groups (Equation (3)). Summing up the areas of all synchronous grafts within the groups we obtained n = 2.5 ± 0.6 (Equation (3) and Figure 5E,F), we took m = 69 as the smallest number of grafts among considered groups. According to the statistical model, the obtained experimental sample was sufficient to identify statistically significant differences; rejection of the null hypothesis gave *p* < 0.001 (Figure 5E). Possible biological and structural heterogeneity of samples was estimated in the statistical model. Values n = 1.9 ± 0.5 and m = 83 could be revised on the assumption that all asynchronized grafts were a consequence of heterogeneity; in this case denial of the null hypothesis gave *p* < 0.001. This estimate maximized potential errors associated with sample properties and measurement inaccuracies. Finally, we get the rejection of the null statistical hypothesis. To sum up, optical mapping experiments showed the increased probability (non-equivalence) of finding stable electrophysiological coupling between suspended iPSC-CMs and confluent cardiac monolayer in the presence of polymer kernels. Thus, we experimentally confirmed our hypothesis that kernels can influence the engraftment of suspended cells into the host tissue.

### 3.4. Analysis of the Assumptions Underlying the Optical Mapping Experiment

**Figure 6:**
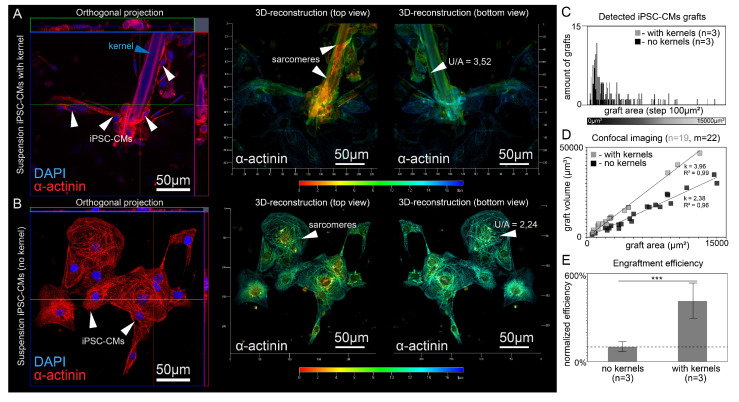
Direct comparison of the iPSC-CMs engrafted with and without fibroin kernels. (**A,B**) Three-dimensional reconstruction of grafts with and without fibroin kernels. (**C**) Area distribution of grafts with successful electrophysiological coupling (synchronized host-graft excitation). (**D**) Graph showing the relationship between U (µm^3^) and A_xy_ (µm^2^) for engrafted iPSC-CMs. Panels (**C,D**) have a common range of horizontal axis. (**E**) Comparison of the efficiency of iPSC-CMs transfer with and without fibroin kernels (n = 3, *** *p* < 0.001 in statistical model) based on conjunction of optical mapping results (**C**) and layer-by-layer confocal microscopy (**D**). The diagram shows the difference in the overall areas of engrafted cells within groups.

In this section, we analyze the result of the last section from two positions: how justified is the estimate of the number of engrafted cells by their area, and whether this area is an additive value that allows summing the areas of all engrafted cells together. To relate the three-dimensional structure of iPSC-CMs to the area they can occupy in recipient monolayer, we performed layer-by-layer confocal imaging of iPSC-CMs on the flat adhesive glass surface (Figure 6A,B). The shape of the cell during adhesion can change significantly, while we consider the cell volume to be a constant value [25,26] that gives an objective assessment of the number of cells and additivity of volume and area in cell clusters. We represented the surface of each cell as H (x, y), a continuous set of points above the glass plane XY (Equations (1) and (2)). Layer-by-layer confocal microscopy of iPSC-CMs with and without polymer kernels determines the values of the cell volume U (µm^3^) and the area occupied by the graft A_xy_ (µm^2^) (Figure 6D). Within each group, there was a linear dependence of U on A_xy_ (in the range from 0 µm^2^ to 15,000 µm^2^ for A_xy_), linear approximation gave R > 0.95 (R = 0.99, n = 19 for cells with kernels and R = 0.96, n = 22 without kernels); approximation coefficients were statistically different (*p* < 0.01, Figure 6D).

(4) $$\forall \;H_{i,}H_{j}\;from\;the\;same\;group\to U(H_{i})+U(H_{j})=k\left(A(H_{i}\right)+A(H_{j}))$$

The presence of the linear dependence (Equation (4)) allowed us to consider the volume of engrafted iPSC-CMs as an additive value determined by its area. Figure 6C shows the areas of grafts recorded by optical mapping, Figure 6D shows the volume and area ratios for adhered iPSC-CMs clusters of the corresponding size. Combination of data from Figure 6C,D allows estimation of the parameter n for statistical model, panel 6F. The estimation adjusted in this way (with the replacement of the measured area of cells by their calculated volume) still gives rejection of the null hypothesis in the statistical model (*p* < 0.001). To sum up, the linear dependence indicates the admissibility of summing the areas of all clusters together, as was done for the Figure 5F and Figure 6E. 

## 4. Discussion

The possibility of iPSC-CMs engraftment with electrical coupling has been demonstrated in various experimental models of intramyocardial injection. However, the control of these processes remains a challenge [3,8]. Our experiments have shown that polymer kernels of subcellular sizes allow suspended iPSC-CMs to bind and form a shell around them, allowing isolated cells to recover the orderly structure of contractile filaments and excitable properties inherent to cells on a regular substrate. Such use of biocompatible polymeric materials made it possible to influence the stochastic process of engraftment of suspended iPSC-CMs into an excitable cell conglomerate. Our experiments showed that excitation-induced Ca^2+^ transients (Figure 5A) in injected cells can correlate with monolayer excitation phases, and the presence of a polymer kernel significantly increases the likelihood of detecting such a correlation.

We show that the presence of a polymer kernel in a cell increases the probability of forming a stable electrophysiological coupling with a monolayer in a direct comparison of two groups of suspension cells, with and without polymer kernels. The presence of polymer kernels is the only difference between the groups (including the expression of the Cx43 connexin that is unchanged, Figure 3G). Therefore, the processes of electrical coupling with a monolayer of an excitable cell with a kernel and a non-excitable cell without a kernel are not equivalent (non-equivalence is expressed by rejection of the null hypothesis in the statistical model). It may be explained by the facilitated coupling scenario between an excitable cell and an excitable monolayer. It has previously been shown that the formation of a contact zone between excitable cells (or cell cultures) leads to 48 h native cardiomyocytes coupling [27] without connexin clustering into gap junctions (GJ) that directly connect intracellular spaces. Electrical conduction in this case is provided by alternative mechanisms, including ephaptic coupling (EpC) [28,29,30]. In addition, the interaction of the recipient tissue with HFN protein that covers the surface of the kernel quickly fixes the relative position of the cells (in 2 h, Video S4) and localizes the contact zone required for co-culturing. This practical implementation of the facilitated coupling can reduce arrhythmogenicity during early engraftment and stabilize the cell ensemble before (or without) GJ coupling. 

We assume that the results described above are also valid for other polymer compositions, since control of the intracellular structure is achieved by the shape of the “adsorbed” substrate and the HFN coating [21]. Fibroin and PLLA were chosen arbitrarily as a base. To the best of our knowledge, these polymers have no specific effect on electrophysiology or the possibility of cell coupling [31,32,33,34]. Restoration of intracellular order turned out to be possible both on nanofiber scaffolds (700–900 nm in diameter, Figure 3A) by the mechanisms described earlier [21], and on large fibroin kernels (19.9 ± 4.9 μm, Figure 2A), which was shown here for the first time. Using the polar transformation of 3D images, it was possible to conclude that both cases are reduced to the curvature of the cell membrane with the enveloping of the polymer kernel (Figure 2A). Therefore, with an increased fiber diameter, no clustering of cells or an effect on their mobility is observed compared to the suspension (Figure 5B, *p* > 0.05). Furthermore, NRVMs has no direct analogue among human-specific cardiac cells due to obvious ethical restrictions and therefore has no clinical application. In this paper, we have shown that the possibility of culturing on kernels is a common feature of NRVMs and human iPSC-CMs (which have promising clinical prospects for the future). The applicability of the NRVMs for culturing on kernels partially follows from the cited paper [21], while the application of human-specific cardiac cells is a novel result.

In the future, large biodegradable kernels could be used in combination with delivered drugs that are released as the polymer biodegrades (including substances from hypoxia [35] and other factors affecting the effectiveness and safety of cell therapy, e.g., immunosuppressants, beta-blockers [36] or if-channel blockers [37]). Nanofiber substrates (Protocol 2) can supply nanoparticles to suspension cells with the least possible use of polymer mass, expanding the potential for creating smart biomaterials. It is of practical interest to use kernels with electrical conductivity and surface roughness, the latter will help increase the surface area with the same linear dimensions of the kernel. 3D-bioprinting with excitable cells will help create multi-layered meshes of cells, avoiding tissue vascularization, especially if the fibers support the shape of a three-dimensional structure.

There are several limitations of the study design. First, we used only one iPSC line reprogrammed from a donor without cardiovascular diseases (CVD). The use of polymer kernels for iPSC lines with mutations associated with CVD and the formation of the contractile apparatus (e.g., hypertrophic cardiomyopathy [23]) will require additional studies. Second, we used a monolayer of iPSC-CMs, which is a simplified two-dimensional model of cardiac tissue. Currently, the creation of a multilayer, significantly more voluminous tissue engineering model, is impossible without a functioning vascular bed [38,39], just as it is impossible to visualize individual cells with optical mapping of three-dimensional samples [40,41], which limits in vitro studies of intramyocardial injections to only a two-dimensional model. The considered stages of engraftment (adhesion, excitability restoration, and electrical coupling with surrounding cells) are mimicked in two-dimensional systems, just as in three-dimensional. Therefore, we believe that mechanism of controlled coupling observed in a two-dimensional model should also work in three-dimensional in vivo models with the reasonable use of laboratory animals. 

An important note is that two of the three kernel dimensions are indeed subcellular. The length of the kernel may exceed the maximum length that the myocyte can spread, although it is of the same order of magnitude. We assume that the use of excessively long kernels would not introduce new negative effects due to cell polarization or separation. Linear length could be an important parameter for kernels made of biodegradable materials, as size determines the duration of decomposition. This issue remains open for further research in in vivo or in vitro experiments with biodegradable polymers. In in vivo experiments, it will be critical to allow the kernels with cells to pass freely through the puncture needle used to deliver the conventional suspension. We used spatiotemporal scans of Ca^2+^ traces visualized with Fluo-4 AM dye (Figure 5A) to study the correlation of excitation-induced Ca^2+^ transients between engrafted cardiomyocytes and a monolayer. The vertical axis in scans shows the change in Fluo-4 AM intensity over time, and the horizontal axis shows the change in space along the selected line (e.g., yellow stripe at Figure 5A) that should intersect both monolayer cells (without CellTracker Red) and injected cells (with CellTracker Red). The presence of a monotonous negative slope on such a scan indicates a unidirectional transmission of excitation from the monolayer to the engrafted cells. Possible artifacts due to the electrotonic effect [42,43] on cell excitation are excluded. Spatiotemporal scans were used to classify cells into synchronized and asynchronized groups based on the presence or absence of electrical coupling with the monolayer. According to the physical meaning, the slope angle tangent is the excitation propagation velocity [44]. However, the compromise of spatial and temporal resolution did not allow revealing the deceleration of the wave that overcomes the engrafted cells. Although such a slowdown is not considered as a sufficient condition for the occurrence of EA [8], it may be a topic for further research. It is interesting to investigate whether the internal anisotropy of cells observed on fiber substrates [15] will help to reduce the delay of the wave during the passage of oriented grafts compared to conventional suspension. In general, we considered the monolayer as an excitable ensemble of cells equally accessible to electrical coupling, and not as a substrate for arrhythmia. For this reason, we did not study the effect of asynchronized clusters on the stability of the excitation wave in the monolayer. 

The statistical model developed by us for the analysis of optical mapping (Equation (3), Appendix B) explicitly takes into account the number of excitable grafts m, whereas the number of monolayers in each group appears implicitly. The limits of applicability of the model are as follows: the entire surface of the monolayer must be equally accessible for cell adhesion (applies to confluent monolayers [45], but not to infarcted hearts, for example [5]), m>>10 to comply with non-strict inequalities. The statistical model made it possible to compare two distributions of synchronized clusters obtained in a series of experiments with polymer kernels (n = 3) and without them (n = 3). Adding asynchronized clusters to the statistical sample is a tool to take into account the inhomogeneities in each group of n monolayers, making an upper estimate. However, the chosen approach to statistics is limited to the analysis of the qualitative differences, confirming only the presence of statistically significant non-equivalence. Thus, the numerical assessment of the contribution of polymer kernels to the probability of coupling will require more experiments and certain changes in the study design, as in this work, we did not track what proportion of cells had time to form a “shell” around the polymer kernel by the time of injection. Taking into account the efficiency of landing on polymer kernels and increasing the number of experiments n (instead of m) would make it possible to understand how the probability of coupling can increase in the presence of a polymer kernel, which may be a task for future in vitro studies. We believe that a direct consequence of the increased likelihood of coupling will be to reduce the side effects of cell therapy, which will allow cell injections with a minimal risk of EA at the early stages of engraftment.

## 5. Conclusions

Our experiments have shown that polymer kernels of subcellular sizes can bind to suspended iPSC-CMs, which form a shell around them, allowing isolated cells to recover the orderly structure of contractile filaments and excitable properties inherent to cells on a full-fledged substrate (e.g., a Petri dish or a polymer matrix). Such use of biocompatible polymeric materials made it possible to influence the stochastic process of engraftment of suspended iPSC-CMs and significantly increase the likelihood of electrophysiological coupling of cells with the recipient tissue.

## Data Availability

Raw data (optical mapping and confocal microscopy) and processed data could be found at: https://drive.google.com/drive/folders/1oueNKWyzOM5g34LL2x85jfjoNSUU72_h?usp=sharing. The iPSC line and original code can be shared upon request to the corresponding author.

## Supplementary Materials

The following supporting information can be downloaded at: https://www.mdpi.com/article/10.3390/mi14010051/s1, Figure S1: SEM micrograph of a fibroin kernel (Protocol 1); Figure S2: Scheme of the experiment to assess the viability of iPSC-CM with and without fibroin kernels; Figure S3: Viability of iPSC-CMs on microcarriers after 4 days without full-fledged substrate; Video S1: Isolated NRVM with adsorbed PLLA kernel, spontaneous mechanical contractions; Video S2: Engrafted NRVM with adsorbed PLLA kernel, spontaneous mechanical contractions with host NRVM monolayer; Video S3: Optical mapping of engrafted iPSC-CMs with fibroin kernels (host iPSC-CMs monolayer) ; Video S4: Adsorption of fibroin kernels by NRVM monolayer (within 2 h).

## Appendix A

Algorithm for processing optical mapping data. Step 1: CellTracker Red signal was subjected to the threshold procedure to obtain the fluorescence boundaries: obtained image was superimposed on the Fluo4-AM signal. To verify the consistency of the excitation between engrafted cells and the recipient, we used procedures, Image/Stacks/Z-axis Profile. Thereafter, Fluo-4AM signal was transformed into a standard deviation map (Image/Stacks/Z-projection) and subjected to the threshold procedure to visualize signal boundaries.

Step 2: Processed Fluo4-AM and CellTracker Red signals were superimposed on each other. In the area of signal crossing, the following criteria were tested: (1) Fluo4-AM and CellTracker Red signals have areas with a common border (2) Fluo4-AM signal does not go beyond the boundaries of the CellTracker Red signal (the opposite is possible).

The resulting markup was used to calculate the area (µm^2^). Lastly, according to the results of step 1, the area was defined as sync (excitation fully synchronized with the recipient tissue) or async (excitation not synchronized).

## Appendix B

Statistical model. The following are the assumptions underlying the statistical model.

Suppose we implant N cells in a monolayer, and we obtain m single embedded (adhered and excited together with the monolayer) cells. We know the area of each cell (s_i_) and the total area of all cells, S. All quantities of s_i_ are normally distributed, values of μ and variance σ are known. The presence of nonzero σ leads to the fact that, when the experiment is repeated, the value of S can vary for the same m (an obvious case, for m = 1).

We can estimate the maximum error in determining the value of s associated with the described variability of the values of s_i_: for this we will act according to the principle of “Maxwell’s demon”. Let us take a large number of cells, from which we randomly take two cells at a time and compare their area. A cell with a larger area will always be placed in dish 1, and one with a smaller area in dish 2 (assuming that the probability of getting two identical cells is zero). After m iterations, we obtain the same number of cells (m) in each dish with the maximum difference in the total areas (S_1_ and S_2_, respectively, S_1_ > S_2_).

Knowing the values of μ and σ, we can relate the probability of obtaining a given n = S_1_/S_2_ with the number of iterations (m). Since s_i_ obeys the normal distribution, the probability of finding a pair of cells with an area difference n time by chance is described as *P* (*A*|*B*):

(A1) $$P\left(A\right)=\overset{a}{\underset{0}{\int }}\frac{1}{\sigma \sqrt{2\pi }}\exp\left(-\frac{{\left(r-\mu \right)}^{2}}{2\sigma^{2}}\right)da^{\prime }$$

(A2) $$P\left(B\right)=\overset{\infty }{\underset{na}{\int }}\frac{1}{\sigma \sqrt{2\pi }}\exp\left(-\frac{{\left(r-\mu \right)}^{2}}{2\sigma^{2}}\right)da^{\prime }$$

Here, event A is manifested as finding the cell with s_i_ < a, and event B is manifested as finding the cell with s_i_ > a*n. The required estimate of P(A|B) for m > 1 iterations is obtained by raising to the power of m >1 (see Discussion section). The desired probability of a random difference between S_1_ and S_2_ by a factor of n depends on the set of values μ, σ, n, m.

(A3) $$P_{\mu ,\sigma ,n}\left(m\right)={\left[\underset{a}{\max}\left(P\left(A|B\right)\right)\right]}^{m},\;P\left(A|B\right)\approx P\left(A\right)P\left(B\right)$$

The maximum of the product of P(A) and P(B) depends on the set of values μ,σ, n (the value a acts as a parameter). All calculations were performed in the Python 3 environment (Jupiter notebook) using the NumPy, PyLab, and sklearn libraries. To find the maximum value of P, we varied the value of a with a step of 0.1 µm^2^. Thus, P = the probability that the comparison of the total areas from one group and from another (e.g. comparison of Figure 5B and Figure 5C) showed a random difference not related to the number of cells in the groups (and, in the context of this work, the efficiency of transplantation). The negation of the null hypothesis (*P* < 0.001), in turn, shows that the resulting difference in total area is possible only if the number of cells in the groups (and hence the transplantation efficiency) is different.
