# Supplementary figures and images for: Polymer Kernels as Compact Carriers for Suspended Cardiomyocytes
Source: Micromachines (Basel). 2022 Dec 25;14(1):51. doi: 10.3390/mi14010051 (PMC9865253; doi:10.3390/mi14010051)

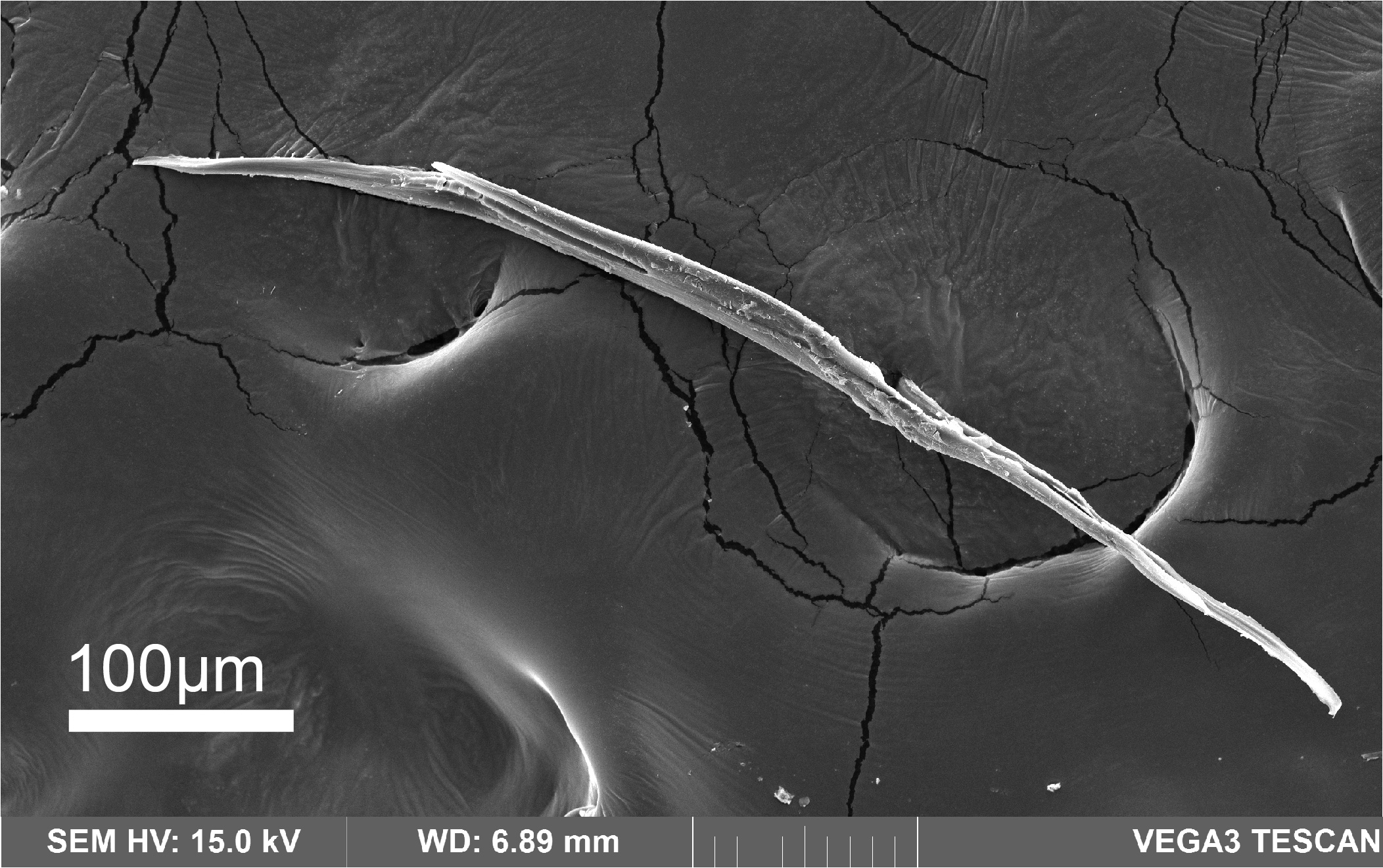

Supplement: Supplementary file 1 [file micromachines-14-00051-s001.zip › Supplementary Figure S1.jpg]

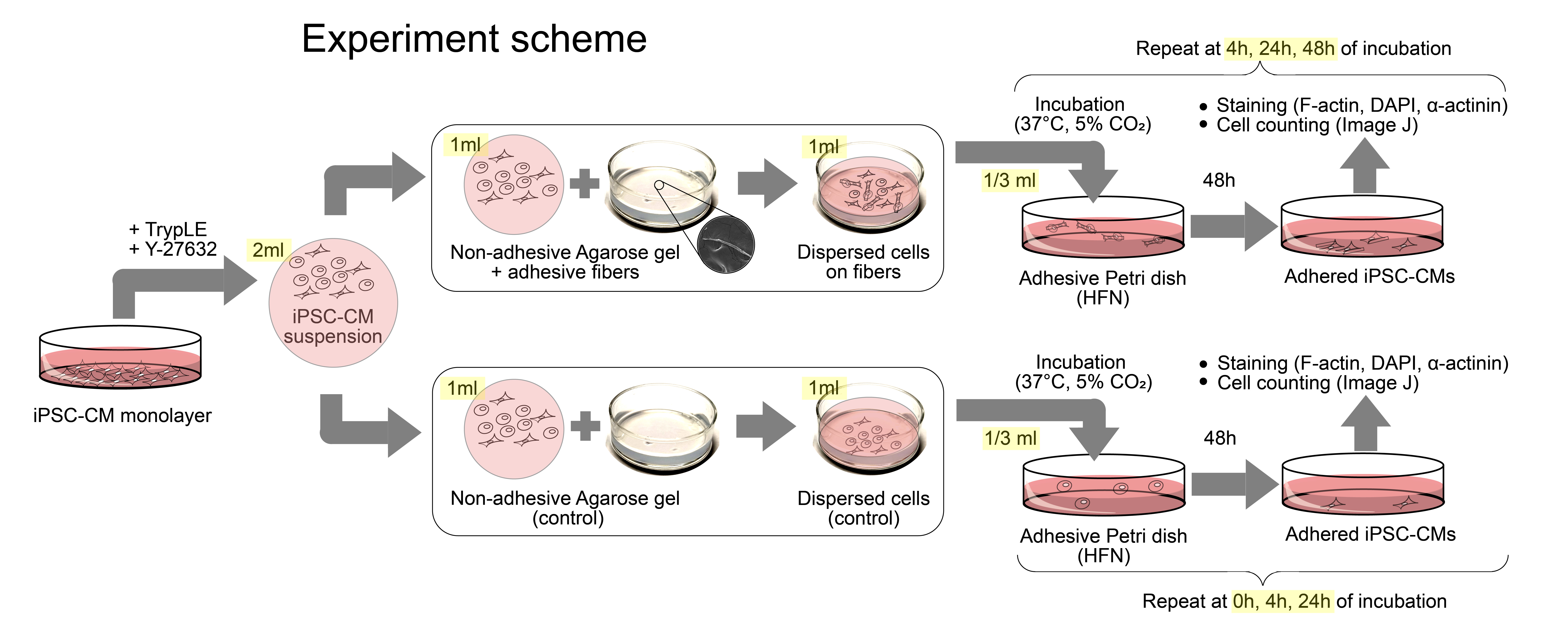

Supplement: Supplementary file 1 [file micromachines-14-00051-s001.zip › Supplementary Figure S2.jpg]

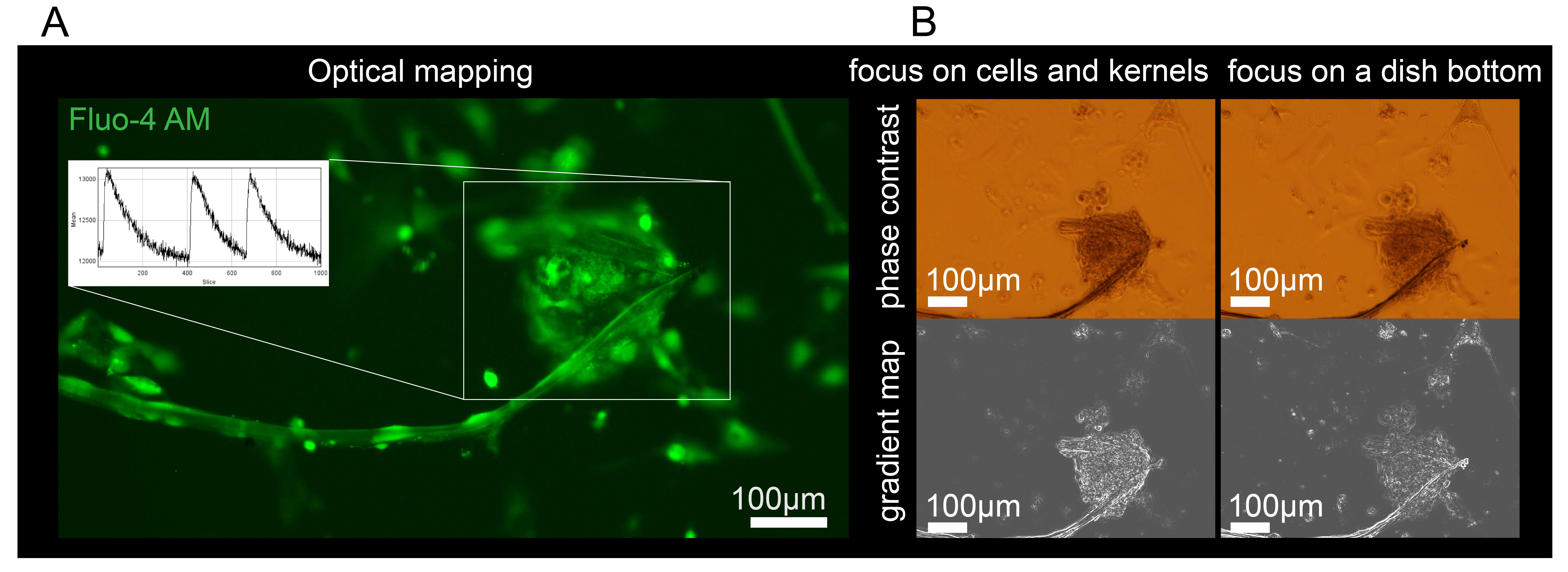

Supplement: Supplementary file 1 [file micromachines-14-00051-s001.zip › Supplementary Figure S3.jpg]
